# Transcriptomic Response of *Rhizobium leguminosarum* to Acidic Stress and Nutrient Limitation Is Versatile and Substantially Influenced by Extrachromosomal Gene Pool

**DOI:** 10.3390/ijms252111734

**Published:** 2024-10-31

**Authors:** Kamil Żebracki, Piotr Koper, Magdalena Wójcik, Małgorzata Marczak, Andrzej Mazur

**Affiliations:** Department of Genetics and Microbiology, Institute of Biological Sciences, Maria Curie-Skłodowska University, Akademicka 19 St., 20-033 Lublin, Poland; kamil.zebracki@mail.umcs.pl (K.Ż.); piotr.koper@mail.umcs.pl (P.K.); magdalena.wojcik2@mail.umcs.pl (M.W.); malgorzata.marczak@mail.umcs.pl (M.M.)

**Keywords:** *Rhizobium leguminosarum*, transcriptomics, nutrient limitation, acidic stress, extrachromosomal replicons (ECRs), pangenome, differential gene expression, acid tolerance, metabolic adaptation

## Abstract

Multipartite genomes are thought to confer evolutionary advantages to bacteria by providing greater metabolic flexibility in fluctuating environments and enabling rapid adaptation to new ecological niches and stress conditions. This genome architecture is commonly found in plant symbionts, including nitrogen-fixing rhizobia, such as *Rhizobium leguminosarum* bv. *trifolii* TA1 (RtTA1), whose genome comprises a chromosome and four extrachromosomal replicons (ECRs). In this study, the transcriptomic responses of RtTA1 to partial nutrient limitation and low acidic pH were analyzed using high-throughput RNA sequencing. RtTA1 growth under these conditions resulted in the differential expression of 1035 to 1700 genes (DEGs), which were assigned to functional categories primarily related to amino acid and carbohydrate metabolism, ribosome and cell envelope biogenesis, signal transduction, and transcription. These results highlight the complexity of the bacterial response to stress. Notably, the distribution of DEGs among the replicons indicated that ECRs played a significant role in the stress response. The transcriptomic data align with the *Rhizobium* pangenome analysis, which revealed an over-representation of functional categories related to transport, metabolism, and regulatory functions on ECRs. These findings confirm that ECRs contribute substantially to the ability of rhizobia to adapt to challenging environmental conditions.

## 1. Introduction

A fraction of currently known bacterial genomes is split between two or more large DNA fragments (over 300 kb), an arrangement referred to as a multipartite or divided genome. This multipartite organization includes a primary chromosome along with a diverse set of secondary, extrachromosomal replicons (ECRs) and is observed in many significant microorganisms, including plant symbionts such as nitrogen-fixing rhizobia [1]. The primary chromosome is always the largest replicon and contains the majority of core or essential genes [2]. In contrast, ECRs show greater variability in sequence and gene content, with a functional bias toward transport, metabolism, and regulatory processes [3,4]. As a result, these replicons are enriched with genes that enable adaptation to various niches, new environments, and stress conditions [5,6,7].

The regulation of gene expression plays a critical role in the cellular response to environmental changes. This is especially evident in the microbial world and is well-illustrated by rhizobia for two main reasons. First, these bacteria are capable of saprophytic growth in soil, and under suitable conditions, they enter symbiotic relationships with compatible plant hosts [8], where they fix nitrogen by converting N_2_ into ammonia. Second, in the soil, rhizobia encounter nutrient-limited and stressful conditions (e.g., low pH, salinity). This environmental complexity seems to be reflected in the structure of rhizobial genomes, which are large and, in most cases, multipartite, containing putative chromids and megaplasmids in various combinations [9]. ECRs in rhizobia can be numerous and large, constituting up to 50% of the total genome size [10,11,12]. Rhizobia appear to benefit from their divided genomes, with the accessory functions encoded by ECRs serving as an extensive toolkit for adapting to challenging environments and stress. Transcriptomic studies on *Sinorhizobium meliloti*, *Rhizobium leguminosarum*, *Rhizobium phaseoli*, and *Rhizobium favelukesii* have demonstrated that genes located on individual ECRs are over-represented among the differentially expressed genes (DEGs) during adaptation to specific niches and stress conditions [13,14,15,16,17].

Environmental stress significantly impacts rhizobia, influencing their growth, survival, and symbiotic relationships with legumes. Various stressors, such as temperature extremes, salinity, pH fluctuations, and heavy metal accumulation, negatively influence rhizobial growth and nodulation, leading to reduced biological nitrogen fixation and lower crop yields. For example, osmotic and temperature stresses, as well as soil acidity or alkalinity, impair nodule formation and function, both of which are essential for nitrogen fixation [18]. Several stress response genes in rhizobia have been shown to play an important role in enabling these bacteria to withstand environmental challenges, thereby improving their symbiotic performance under adverse conditions [19]. Recent studies suggest that the mechanisms rhizobia developed to tolerate environmental stress have been co-opted by both plant and bacterial partners during symbiosis, facilitating the successful exchange of signals required for effective nitrogen fixation [20].

Nutrient deficiency and extracellular acidity significantly hinder the growth of rhizobia in soil and limit their ability to form nitrogen-fixing symbioses with legume hosts [21]. As nutrient limitation affects both the free-living and symbiotic forms of rhizobia, it may be expected to trigger a complex reprogramming of gene expression across different genome compartments. A global transcriptomic study on *Rhizobium etli* revealed that nutrient limitation induces significant reprogramming of gene expression, primarily through the (p)ppGpp signaling pathway [22]. However, there is considerable variation in how different rhizobia genera, species, and even strains respond to nutrient deficiencies [23]. The effects of nutrient deficiency on free-living rhizobia, especially from a global transcriptomic perspective, remain poorly understood [24].

Soil acidity also impairs the free-living growth of rhizobia and their nitrogen-fixing symbiosis with legumes, which has significant agronomic importance [21]. Several mechanisms have been identified in rhizobia that help them sense and respond to external acidic pH. In *S. meliloti* and *Agrobacterium tumefaciens*, the physiological responses to acidity involve the differential expression of genes related to exopolysaccharide (EPS) biosynthesis, chemotaxis, motility, central carbon metabolism, and respiration [21,25,26]. The *S. meliloti* chromosome was found to be the replicon with the highest density of acid-induced genes [21]. In *Mesorhizobium loti*, the acid response includes the altered expression of several genes related to ABC transporters and cell envelope proteins [27]. In the acid-tolerant strain *R. favelukesii*, which can withstand exposure to pH 4.6, 1924 genes were differentially expressed under acidic conditions, with around 60% being downregulated. The transcriptomic response in *R. favelukesii* mainly involved changes in the expression of genes related to energy metabolism and protein turnover, as well as mechanisms like GABA and histidine metabolism, cell envelope modifications, and reverse proton efflux. DEGs were distributed across all replicons (the chromosome and four ECRs), with approximately 50% located on the chromosome. Notably, 60% of the coding sequences (CDSs) on the symbiotic plasmid were underexpressed, and a derivative strain cured of this plasmid showed improved performance under acidic conditions [17]. In *Rhizobium tropici* CIAT 899, 383 genes were differentially expressed under acidic conditions, with only 11 genes induced during acid shock. The acid stress response was versatile, involving altered expression of genes encoding response regulators, membrane transporters, enzymes involved in amino acid and carbohydrate metabolism, and proton extrusion [28]. Interestingly, the overexpression of genes commonly associated with acid tolerance in rhizobia, such as *act* and *exo*, was not universally detected across different species [27]. Therefore, the current model of acid tolerance in rhizobia encompasses multiple systems that sense and respond to pH changes in diverse ways. This reflects the complexity of the acid stress response and highlights the need for further research into novel mechanisms of bacterial adaptation to environmental pH changes. It has been established that many ecologically significant processes are differentially mediated by prokaryotes, even at the strain level [29].

*R. leguminosarum* bv. *trifolii* TA1 (RtTA1) is the symbiont of *Trifolium* spp. and has a long history of use as a commercial inoculant for perennial and annual clovers. Furthermore, this strain has been adopted by the international community as a model organism to investigate the biology of the *Trifolium–Rhizobium* symbiosis [30]. The RtTA1 genome consists of five replicons—a chromosome and four ECRs (named pRleTA1a–d)—ranging in size from 476 to 808 kb, designated as both chromids and megaplasmids [31]. RtTA1 ECRs were shown to confer significant metabolic versatility to the bacteria [32]. In *R. leguminosarum* bv. *trifolii*, strains sensitive to low pH have been found to possess an altered proton-permeable membrane and, in some cases, reduced proton release activity compared to pH-tolerant strains [33]. However, a comprehensive transcriptomic analysis of *R. leguminosarum* in response to nutrient limitation and acidic conditions has not yet been conducted.

In this study, we performed transcriptome analyses on RtTA1 cells grown under stressful conditions, including partial nutrient limitation and low pH, using high-throughput RNA sequencing (RNA-seq). The response of RtTA1 to the applied stressful conditions was versatile and complex, with each tested growth variant leading to significant changes in the expression levels of numerous genes. The resulting data revealed both similarities and differences with other rhizobia in response to stress conditions, demonstrating the importance of such studies in species more distant from typical model organisms, and highlighting the role of the accessory genome in stress adaptation.

## 2. Results and Discussion

### 2.1. The ECR Pool in the Rhizobium Genus Is Enriched in COG Categories Related to Carbohydrate and Amino Acid Transport and Metabolism, Transcription, and Signal Transduction

**Figure 1:**
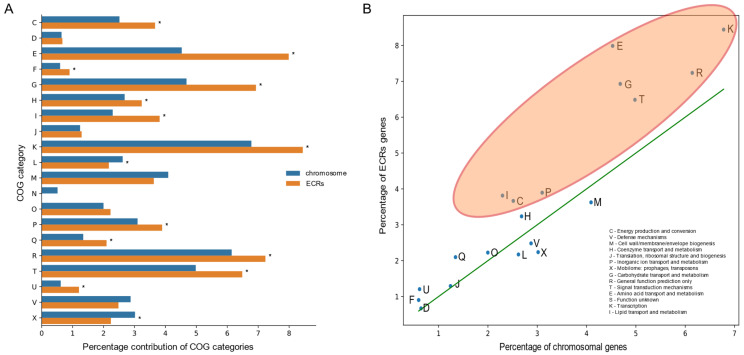
Percentage distribution of individual COG categories in the accessory pangenomes of chromosomes and plasmids from *Rhizobium* genomes. (**A**) Bar chart showing the percentage share of COG categories in the analyzed pangenomes, with statistically significant differences (*p* value < 0.05) marked by an asterisk. (**B**) Scatter plot showing the relationship between the percentage of individual COG categories in the accessory pangenomes of chromosomes (*x*-axis) and plasmids (*y*-axis). The 1:1 line was shown in green. COG categories significantly more abundant in plasmid pangenome are marked with a red ellipse.

To better understand the role of ECRs in *Rhizobium* adaptation to environmental stress, we began by examining the distribution of functional COGs (clusters of orthologous genes) categories across chromosomes and ECRs within the entire *Rhizobium* genus. Using complete genome sequences available in GenBank, we constructed and analyzed the *Rhizobium* pangenome (as described in the Materials and Methods section) (Figure 1A). Several functional COG categories, particularly those associated with carbohydrate and amino acid transport and metabolism (COGs G, E), transcription (COG K), signal transduction (COG T), and general functions (COG R), were found to be over-represented in the *Rhizobium* pangenome. Notably, extrachromosomal replicons were significantly enriched with genes from most of these COG categories (Figure 1B).

The over-representation of genes belonging to COG categories associated with environmental adaptation in the *Rhizobium* ECR genome supports the hypothesis that extrachromosomal replicons play a significant role in adaptation to ecological niches and stress responses. This suggests that ECRs are a fundamental evolutionary factor shaping the functional profile of *Rhizobium*. Importantly, the pangenome analysis provided a foundational perspective on how ECRs contribute to key biological processes such as nutrient transport, metabolism, transcriptional regulation, and signal transduction. By establishing the functional enrichment of ECRs in various functional categories, we aimed to contextualize their involvement in the broader adaptive strategies of *Rhizobium* species, laying the groundwork for more detailed transcriptomic analyses of RtTA1 under stress conditions.

### 2.2. Transcriptome Response of R. leguminosarum Under Acidic Conditions and Nutrient Limitation

The transcriptomes were sequenced and quantified as described in the Materials and Methods section. For individual samples, 13,654,634 to 44,837,078 high-quality paired-end reads were obtained, with 88–99% of the reads mapped to the reference genome of *R. leguminosarum* bv. *trifolii* TA1 (Supplementary Table S1). Of the 7028 CDSs annotated in the RtTA1 genome, 6945 to 6975 genes were represented in this RNA-seq study across the different growth conditions (Supplementary Tables S2–S5). A key aspect of the RNA-seq analysis was identifying DEGs whose expression levels changed significantly under the tested conditions (Supplementary Table S6). Differential gene expression was compared across four experimental conditions: (1) M1 minimal medium vs. 79CA complete medium at pH 7 (M7vC7), (2) M1 minimal medium vs. 79CA complete medium at pH 5 (M5vC5), (3) pH 5 vs. pH 7 in 79CA complete medium (C5vC7), and (4) pH 5 vs. pH 7 in M1 minimal medium (M5vM7) (Table 1). These abbreviations (M7vC7, M5vC5, C5vC7, and M5vM7) will be used throughout this manuscript to refer to the different experimental conditions. During the growth of RtTA1 under these conditions, 1035 to 1700 genes (14.7–24.2% of the total RtTA1 CDSs) were found to be significantly differentially expressed (Table 1), with downregulated genes predominating in all comparisons except M7vC7.

**Figure 2:**
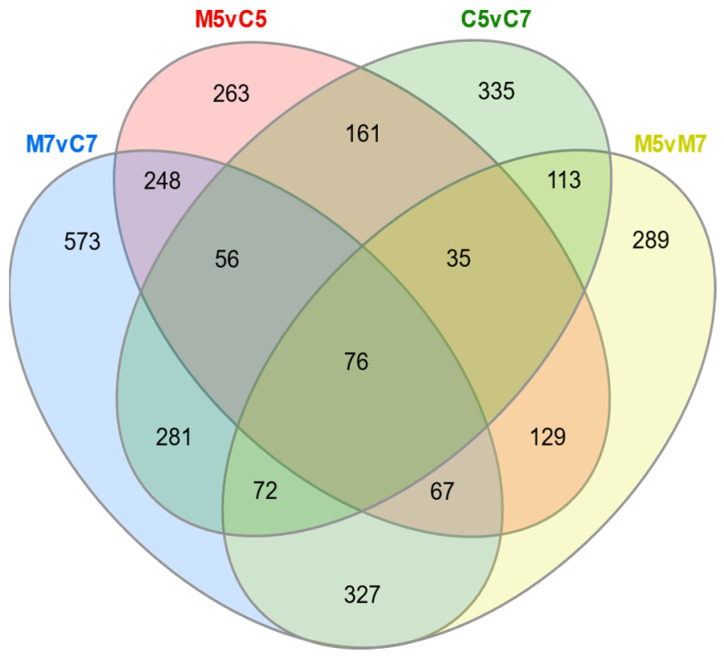
Venn diagram illustrating the distribution of DEGs shared among the experimental conditions tested for *R. leguminosarum* bv. *trifolii* TA1.

A Venn diagram analysis revealed that 447 genes were involved in the response to nutrient limitation, independent of pH, while 296 genes were differentially expressed in response to acidic pH, regardless of the growth medium (Figure 2). A large proportion of these DEGs (87.25%) exhibited consistent behavior (either upregulation or downregulation) in the transition from minimal to complete medium, irrespective of pH (M7vC7 and M5vC5 comparisons; Supplementary Table S7). Similarly, 78.72% of common DEGs showed consistent expression changes between pH 5 and neutral pH, independent of the medium (M5vM7 and C5vC7; Supplementary Table S8). This correlation in gene expression patterns might suggest common regulatory mechanisms governing responses to both pH changes across different media and nutrient limitations, regardless of pH. The analysis also highlighted the broad scope of bacterial responses to varying environmental conditions, as evidenced by numerous DEGs unique to specific transitions—1253 and 588 DEGs in M7vC7 and M5vC5, and 833 and 812 DEGs in C5vC7 and M5vM7 experimental variants, respectively (Figure 2). The relatively high proportion of DEGs with consistent expression patterns points to robust regulatory mechanisms that enable bacteria to adapt efficiently to changes in nutrient availability and pH. However, the presence of condition-specific DEGs indicates that bacteria also employ a variety of specialized responses to fine-tune their physiological adaptation, optimizing survival under distinct environmental stresses.

Additionally, a common pool of 76 DEGs responded to both pH stress and nutrient limitation (Figure 2). The transcriptional profiles of these 76 DEGs were diverse across all tested conditions (Supplementary Table S9), with only 7 genes showing consistent modes of expression (upregulation): *RLTA1_RS14490* (ketol-acid reductoisomerase IlvC), *RLTA1_RS16360* (glutamine synthetase), *RLTA1_RS21785* (acyl-CoA/acyl-ACP dehydrogenase), *RLTA1_RS01905* (flagellin protein FlaA), *RLTA1_RS21625* (ammonium transporter), *RLTA1_RS08365* (nitrite reductase small subunit NirD), and *RLTA1_RS24970* (substrate-binding protein of ABC transporter complex for basic amino acids, glutamine, opines). Furthermore, of the 76 core DEGs, 27 were located on ECRs, with the majority (15 DEGs) found on the pRleTA1a plasmid. This suggests that the response of RtTA1 cells to the applied stressful growth conditions involves both chromosomal and extrachromosomal compartments, highlighting the role of ECRs in stress adaptation.

### 2.3. Pattern of DEG Distribution Among RtTA1 Replicons—ECRs Contribute Significantly to Stress Response

**Figure 3:**
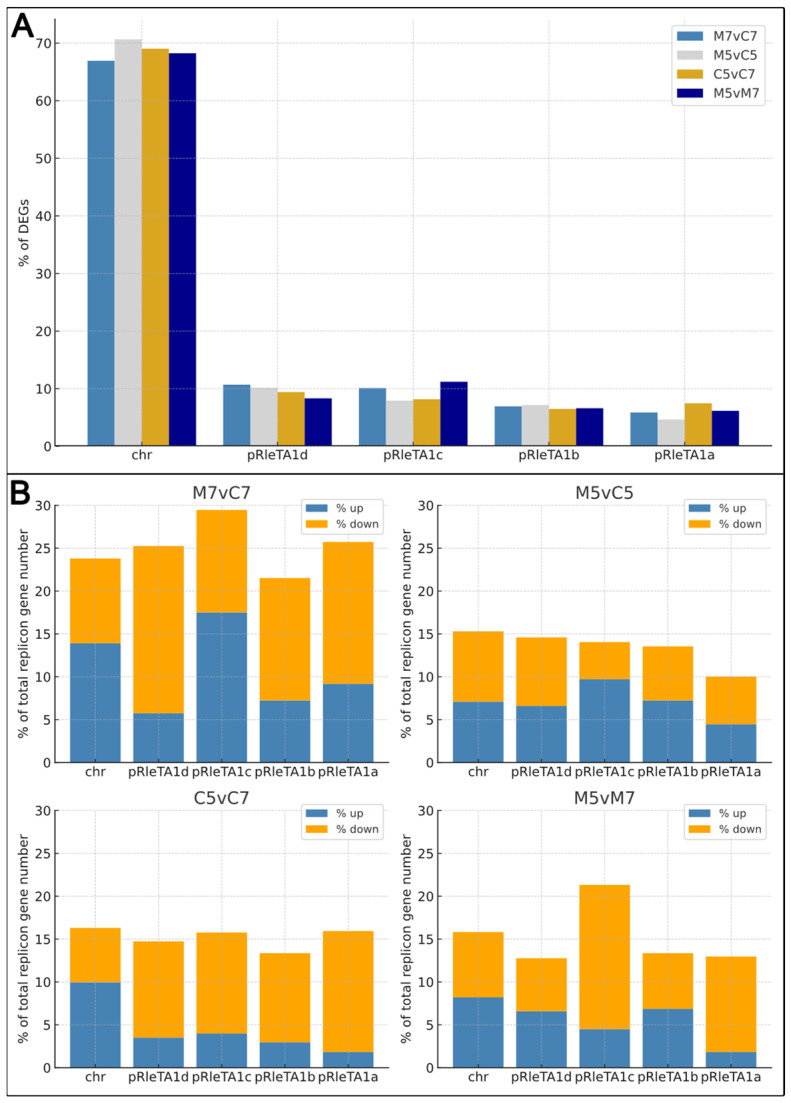
Differential expression analysis and genomic distribution of DEGs in *R. leguminosarum* bv. *trifolii* TA1 under various growth conditions. (**A**) The percentage of DEGs in each replicon, relative to the total number of DEGs in the whole genome, is plotted for each experimental condition. (**B**) Genomic distribution of DEGs across individual replicons of *R. leguminosarum* bv. *trifolii* TA1 under the tested growth conditions. For each replicon, the percentage of DEGs relative to the total number of CDSs is shown, with downregulated (orange) and upregulated (blue) genes indicated. The panels correspond to the experimental conditions described above.

The DEGs were not evenly distributed across the RtTA1 replicons under the tested growth conditions, with most DEGs located on the chromosome (Figure 3A; Supplementary Table S10). However, after normalizing the number of DEGs to the total number of CDSs in each replicon, it became evident that the ECRs played a significant role in the transcriptomic response of RtTA1 to environmental stresses (Figure 3B). The proportion of DEGs relative to the total number of genes in each replicon showed that individual RtTA1 ECRs contributed comparably or even more than the chromosome under certain conditions. For example, growth in minimal medium at neutral pH (M7vC7) led to a high proportion of DEGs in pRleTA1d, pRleTA1c, and pRleTA1a. In contrast, growth under acidic conditions, especially in minimal medium (M5vM7), triggered strong gene expression reprogramming on pRleTA1c (Figure 3B).

The rate of differential gene expression (DEG rate) also varied between replicons (Supplementary Table S11). The chromosome generally showed a higher median log_2_ fold change (log_2_FC) compared to the ECRs, indicating that genes on the chromosome tended to exhibit stronger upregulation across experimental conditions. Meanwhile, pRleTA1a and pRleTA1d often displayed the most pronounced downregulation (more negative mean and median log_2_FC values), suggesting that genes on these replicons were more likely to be suppressed under the tested conditions. In contrast, pRleTA1b and pRleTA1c showed more variable DEG rates. On average, DEGs on pRleTa1c were upregulated under nutrient limitation conditions, irrespective of pH. Overall, the DEG rate analysis provides insight into how genes located on different RtTA1 replicons respond to environmental stresses and nutrient limitations. The uneven distribution of DEGs and variations in DEG rates among replicons underscore the specialized roles of chromosomal and ECR-encoded genes in bacterial adaptation.

### 2.4. Assignment of DEGs in RtTA1 Grown Under Stress Conditions to COG Categories and KEGG Pathways

**Figure 4:**
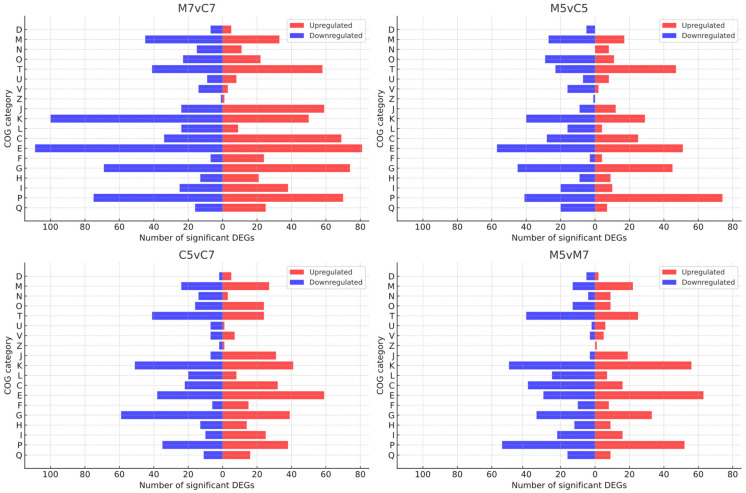
Representation of COG categories comprising DEGs of *R. leguminosarum* bv. *trifolii* TA1 under stress conditions. The COG categories include cellular processes and signaling group: D—cell cycle control, cell division, chromosome partitioning, M—cell wall/membrane/envelope biogenesis, N—cell motility, O—post-translational modification, protein turnover, chaperones, T—signal transduction mechanisms, U—intracellular trafficking, secretion, and vesicular transport, V—defense mechanisms, Z—cytoskeleton; information storage and processing group: J—translation, ribosomal structure, and biogenesis, K—transcription, L—replication, recombination, and repair; metabolism group: C—energy production and conversion, E—amino acid transport and metabolism, F—nucleotide transport and metabolism, G—carbohydrate transport and metabolism, H—coenzyme transport and metabolism, I—Lipid transport and metabolism, P—inorganic ion transport and metabolism, Q—secondary metabolites biosynthesis, transport, and catabolism. The panels correspond to the experimental conditions described above.

To identify the metabolic pathways and biological processes modulated in *R. leguminosarum* cells in response to stress, DEGs were functionally annotated using COG categories and KEGG metabolic pathways (Supplementary Table S6). Across the different growth conditions, the DEGs were distributed into 19 of the 26 COG functional categories. The proportion of upregulated and downregulated DEGs within each COG category is summarized in Figure 4.

A significant portion of the DEGs (30.88%, 36.04%, 36.49%, and 39.89% in the M7vC7, M5vC5, C5vC7, and M5vM7 experimental variants, respectively) represented genes with unknown functions. This finding indicates that a substantial number of genes involved in the stress response of *R. leguminosarum* under the tested conditions have not yet been characterized. These uncharacterized DEGs could potentially play novel roles in adaptation to acidic conditions and nutrient limitation, underscoring the need for further investigation to determine their specific contributions to stress tolerance and metabolic reprogramming [34].

### 2.5. COG and KEGG Categories for Up- and Downregulated Genes: pH 5 vs. pH 7

Under low pH conditions, upregulated genes were primarily found in COG E (amino acid transport and metabolism), COG J (translation, ribosomal structure, and biogenesis), and COG M (cell wall/membrane/envelope biogenesis). In contrast, COG G (carbohydrate transport and metabolism), COG T (signal transduction, including chemotaxis-related pathways), and COG L (replication, recombination, and repair) were dominated by downregulated genes. COG K (transcription) and COG P (inorganic ion transport and metabolism) also showed significant representation among DEGs in RtTA1 grown at acidic pH, though the upregulation and downregulation patterns within these categories varied depending on the medium (Figure 4).

### 2.6. Production and Consumption of Ammonia as a Key Mechanism to Cope with Acid Stress

Based on KEGG pathway analysis, the upregulated genes in RtTA1 classified under COG E were primarily related to the biosynthesis of amino acids such as glutamine, arginine, serine, threonine, lysine, cysteine, and ornithine. Among the most strongly upregulated genes were those encoding glutamine synthetase (*RLTA1_RS16360*), along with two components of glutamine ABC transporters located on plasmid pRleTA1d: the permease (*RLTA1_RS24975*) and ATP-binding protein GlnQ (*RLTA1_RS24985*), 4-aminobutyrate:2-oxoglutarate aminotransferase (*RLTA1_RS23025*), involved in alanine, aspartate, and glutamate metabolism using pyridoxal 5′-phosphate as a cofactor [35], threonine synthase (*RLTA1_RS03510*), urea ABC transporter components UrtABC (*RLTA1_RS17340*, *RLTA1_RS17335*, *RLTA1_RS17330*), argininosuccinate synthase ArgG (*RLTA1_RS13435*) and lyase ArgH (*RLTA1_RS20445*), and N-acetyl-gamma-glutamyl-phosphate reductase ArgC (*RLTA1_RS06990*). Glutamine is a crucial metabolite in bacterial physiology, essential not only for protein biosynthesis but also for the production of various nitrogen-containing compounds [36]. Arginine, a nitrogen-rich amino acid, is similarly important, serving as both a common protein precursor and a potential carbon and energy source for many bacteria [37]. Additional upregulated DEGs included genes coding for ATP phosphoribosyltransferase HisZ (*RLTA1_RS02615*), which is required for the first step of histidine biosynthesis [38], aromatic amino acid aminotransferase HisC (*RLTA1_RS20520*), cysteine synthase CysK (*RLTA1_RS24485*), serine hydroxymethyltransferase (*RLTA1_RS06695*), and ornithine cyclodeaminase ArcB (*RLTA1_RS13975*), all of which were induced under low pH conditions. Genes involved in branched-chain amino acid metabolism, such as leucine-specific ABC transporter (*RLTA1_RS16300*) and leucine transaminase (*RLTA1_RS14465*), were also strongly upregulated.

The upregulation of amino acid metabolism genes is a well-documented bacterial response to pH stress. For example, increased histidine levels in *R. favelukesii* LPU83 have been linked to acid stress, alongside the induction of genes such as *gltD*, *glnT*, and *glsA*, involved in alanine, aspartate, and glutamate metabolism [17]. In line with the observed increase in ammonium turnover under acidic stress in RtTA1, significant upregulation was noted for several genes encoding nitrate reductase subunits, including *nirB* (*RLTA1_RS08370*), *nirD* (*RLTA1_RS08365*), and *nasA* (*RLTA1_RS08360*). Additionally, genes from the COG P category, coding for nitrate/nitrite ABC transporter components [39], such as *narK* (*RLTA1_RS08375*), *RLTA1_RS08380*, and pRleTA1c-encoded *nrtABC* (*RLTA1_RS30500*, *RLTA1_RS30505*, *RLTA1_RS30510*), were upregulated. The genes *narK* (*RLTA1_RS08375*)*, nirB, nirD*, and *nasA* likely constitute an operon, and they exhibit similar expression patterns. The log_2_FC values for these genes were 6.72, 6.93, 7.38, and 4.79 in the M5vC5 growth variant and 7.00, 6.87, 6.50, and 4.86 in M5vM7, indicating strong upregulation and suggesting that both low pH and minimal medium are required for operon activation. The absence of operon induction at neutral pH (M7vC7) emphasizes the importance of this mechanism in response to acid stress, rather than nutrient limitation alone. Dissimilatory nitrite reductases, which catalyze the reduction of nitrate to nitrite, play a critical role in the nitrogen cycle. These enzymes participate in microbial denitrification and nitrate/nitrite ammonification—two catabolic pathways that compete for nitrate utilization depending on environmental conditions. Carbon-limited conditions favor denitrification, leading to nitrite accumulation, whereas carbon-rich environments promote ammonification [40].

The upregulation of amino acid biosynthesis genes aids bacteria in coping with acid stress by enhancing their ability to produce essential metabolites such as glutamine and arginine. This response, coupled with increased ammonia production, supports efficient nitrogen cycle management in challenging environments.

### 2.7. Acidic pH Stress Induces Significant Protein Turnover

Under acidic conditions, almost all DEGs in the COG J category, which corresponds to translation, ribosomal structure, and biogenesis, were upregulated. This category included a large group of genes encoding structural ribosomal proteins and rRNA-binding proteins (*rpmB*, *rpsP*, *rpmA*, *rpsF*, *rpsL*, *rpsG*, *rplX*, *rpsN*, *rplF*, *rplN*, *rplD*, *rpsH*, *rplE*, *rplB*, *rpsI*, *rplM*), as well as genes involved in aminoacyl-tRNA synthetase activity and tRNA modification (*gatA*, *gatC*, *trmB*, *mnmC*, *mtaB*) [41]. Additionally, genes encoding ribonuclease D (an exonuclease involved in the 3′ processing of precursor tRNAs) and translation elongation factors (*tuf, tsf, fusA*) were upregulated.

These findings suggest that increased translational activity and a high rate of protein turnover are part of a coordinated cellular response to maintain integrity and functionality under acidic stress. By synthesizing new proteins to replace damaged ones, bacteria can adapt and survive in acidic environments. Furthermore, the strong upregulation of genes involved in protein repair and degradation supports this conclusion. Specifically, COG O contained upregulated genes encoding hypothetical chaperones, including GroEL (*RLTA1_RS02640*), GroES (*RLTA1_RS02645*), HtpG (pRleTA1a-encoded *RLTA1_RS35010*), and HtrA family protease (*RLTA1_RS35010*), a family of chaperones and serine proteases. These systems play crucial roles in regulating stress responses and maintaining protein quality in the bacterial periplasm [42]. Their upregulation reflects the increased demand for protein synthesis and quality control mechanisms during acid stress.

### 2.8. The Cell Envelope Is Crucial for Protecting Cells Against Harmful Environmental Conditions and Acts as a Barrier to Stress

In *R. leguminosarum*, a number of genes related to cell wall, membrane, and envelope biogenesis (COG M) were upregulated under acidic conditions. These genes are involved in processes such as peptidoglycan synthesis and remodeling. For example, two genes (*RLTA1_RS11670* and *RLTA1_RS03290*) coding for *dacF* family D-alanyl-D-alanine carboxypeptidases (DD-CPases) were upregulated, as well as genes encoding a putative lytic transglycosylase Ltg (*RLTA1_RS15140*) and membrane-bound lytic murein transglycosylase MltA (*RLTA1_RS22530*). MltA catalyzes the cleavage of the β-1,4-glycosidic bond between N-acetylmuramic acid and N-acetylglucosamine in peptidoglycan, a critical process for bacterial cell growth and division [43]. The cleavage of glycosidic bonds by Ltg facilitates the insertion of peptidoglycan precursors during cell growth and division, as well as the construction of membrane-spanning structures, such as flagella and secretion systems [44]. Many bacteria alter their envelope in response to acidic environments, e.g., in *A. tumefaciens*, 17 genes functionally related to the cell envelope was induced in cells grown at pH 5.5 [25].

Additionally, several upregulated genes in the COG M category were involved in the biosynthesis of outer membrane components, particularly lipopolysaccharide (LPS). These include *wcaJ* (*RLTA1_RS06950*), encoding a sugar transferase involved in LPS synthesis, *kdsB* (*RLTA1_RS23435*), encoding 3-deoxy-manno-octulosonate cytidylyltransferase, and *kdtA* (*RLTA1_RS02725*). KdsB activates KDO (3-deoxy-D-manno-octulosonic acid) for incorporation into bacterial LPS in Gram-negative bacteria, while KdtA is responsible for the transfer of KDO to lipid A of LPS [45]. Other upregulated genes involved in lipid A biosynthesis include *msbB* (*RLTA1_RS12505*, coding for lauroyl acyltransferase), *yiaD* (*RLTA1_RS14820*, encoding a putative OmpA family outer membrane porin), and *rfbB* (*RLTA1_RS06725*, coding for dTDP-glucose 4,6-dehydratase, which participates in O-antigen synthesis). Previous studies have highlighted the role of RfbB in maintaining cell wall integrity, cellular morphology, motility, and virulence in bacteria [46]. The composition and structure of LPS in Gram-negative bacteria are highly dynamic, undergoing significant remodeling in response to environmental changes [47,48].

Interestingly, the downregulation of *ropAch1* (*RLTA1_RS12315*), which encodes a putative porin protein involved in the passive diffusion of small hydrophilic molecules, supports the hypothesis that rhizobia specifically modify their cell envelope under acidic stress to reduce membrane permeability, forming a tighter barrier against the entry of H^+^ ions. Similar changes in membrane permeability have been previously reported in *R. favelukesii* LPU83, suggesting that such envelope modifications may play a role in acid stress resistance [17].

In addition to structural proteins of the cell envelope, surface polysaccharides may also be altered during acid stress. A correlation between EPS production and acid response is known in *S. meliloti* and *A. tumefaciens* [25,26]. However, in *R. favelukesii*, the amount of EPS produced under acidic treatment was similar to that obtained under neutral conditions [17]. Although no significant differential expression was observed in genes directly involved in EPS biosynthesis (e.g., the Pss-I gene cluster [49]), changes in surface polysaccharide production were detected in RtTA1 under the tested conditions (Supplementary Figure S1). Nevertheless, some genes related to the biosynthesis of polysaccharides were upregulated, such as a putative *cpsF* (*RLTA1_RS02020*), likely involved in capsular polysaccharide biosynthesis, and three putative glycosyltransferase genes (*RLTA1_RS24165*, *RLTA1_RS05925*, *RLTA1_RS05930*), which may contribute to the synthesis of various surface glycoconjugates.

Based on the observed expression of LPS and EPS biosynthesis genes, it can be concluded that under acidic stress conditions, the protective mechanism in *R. leguminosarum* does not primarily rely on increased EPS production. Instead, the bacteria appear to strengthen the cell envelope by upregulating the biosynthesis of LPS components, thereby forming a more effective barrier against H^+^ ions entry.

### 2.9. Numerous Downregulated Genes Under Low pH Are Linked to Carbohydrate Transport and Metabolism, Signal Transduction, and DNA Replication, Recombination, and Repair

Under acidic conditions, 79 and 51 DEGs related to carbohydrate transport and metabolism (COG G) were identified in RtTA1 when grown in complete and minimal media, respectively. Downregulated genes predominated in this category, particularly those encoding ABC transporter components involved in the transport of monosaccharides (e.g., ribose) and oligosaccharides (e.g., raffinose), as mapped by the KEGG pathway. Several upregulated genes in this category are noteworthy. Increased levels of enzymes from the pentose phosphate pathway (PPP) have been observed in rhizobia exposed to acid stress [50]. The use of carbon through the PPP in stress conditions may indicate an increased need for metabolic intermediates. In our study, *tktA* and *tktB* (*RLTA1_RS12020* and *RLTA1_RS12025*), encoding transketolase domains participating in the non-oxidative branch of the PPP [51], were strongly induced (log_2_FC > 2.5). Additionally, *gnd* (*RLTA1_RS12480*), encoding a putative NADP-dependent phosphogluconate dehydrogenase involved in the oxidative branch of the PPP, was upregulated. This enzyme catalyzes the oxidative decarboxylation of 6-phosphogluconate to ribulose-5-phosphate, producing NADPH [51]. Upregulation of glucose-6-phosphate isomerase (*RLTA1_RS00725*), which catalyzes the second step in glycolysis, was also observed. These findings suggest a higher energy demand in rhizobia exposed to acidic stress.

Further evidence of increased energy demand is seen in the upregulation of genes involved in oxidative phosphorylation, predominantly assigned to COG C, as observed in other rhizobia under low pH stress [21,25,52]. In *R. leguminosarum*, this included the upregulation of *cycF* (*RLTA1_RS05295*, coding for a putative cytochrome C component), genes encoding components of the ubiquinol-cytochrome C reductase complex (*RLTA1_RS16015* and *RLTA1_RS16020*) which generates an electrochemical potential for ATP synthesis, *RLTA1_RS20970* (coding for cytochrome b561), and *cyoB* and *cyoC* (*RLTA1_RS31510* and *RLTA1_RS31505*), encoding cytochrome O ubiquinol oxidase components. Furthermore, the upregulation of isocitrate dehydrogenase (*RLTA1_RS11540*), a key enzyme in the oxidative PPP, Entner–Doudoroff (ED) pathway, and tricarboxylic acid (TCA) cycle, underscores the importance of NADPH generation [53]. Though COG C genes were not highly represented in this study, their upregulation suggests their role in energy acquisition and respiration in *R. leguminosarum* during acid stress.

In the signal transduction category (COG T), downregulated genes predominated. KEGG analysis mapped many silenced kinases-coding genes to pathways related to chemotaxis. The expression profile of RtTA1 COG N (chemotaxis) DEGs varied depending on the growth medium: downregulated genes prevailed during the growth in complete medium, while in the minimal medium, induced genes were mainly observed (Figure 4). This aligns with previous studies showing that motility and chemotaxis respond differently depending on bacterial species and environmental conditions, with reduced motility under acid stress in *S. meliloti*, *R. leguminosarum* bv. *viciae*, and *A. tumefaciens* [25,26,54]. However, some COG T DEGs (also located in the RtTA1 ECR pool), such as genes encoding putative two-component transcriptional response regulators (e.g., LuxR family) and sigma factors, were upregulated in our study. According to the KEGG mapper, the induced regulators could be involved in nitrogen metabolism, such as the nitrogen-specific signal transduction histidine kinase NtrB (*RLTA1_RS09690*), which correlates with the previously described predominance of upregulated genes in the COG E category. Additionally, two genes encoding proteins with EAL domains of bifunctional diguanylate cyclase/phosphodiesterase [55] (located on the chromosome and pRleTA1d) were upregulated, pointing to a potential role for cyclic di-guanosine monophosphate (c-di-GMP) in regulating RtTA1 functions under stress. The ubiquitous c-di-GMP is an important messenger for the control of many bacterial cellular functions, including virulence, motility, cellulose biosynthesis, adhesion, secretion, community behavior, biofilm formation, and cell differentiation. The synthesis of c-di-GMP is triggered by external stimuli acting on various signaling domains within the N-terminal region of a diguanylate cyclase [56].

In the COG L category (replication, recombination, and repair), downregulated genes also predominated (e.g., rec*A*, *dnaA*). However, the expression of a uracil-DNA glycosylase coding gene (*RLTA1_RS16975*) and several genes encoding putative DNA methyltransferases—*RLTA1_RS09075* (DNA cytosine methyltransferase), *RLTA1_RS03560* (belonging to *ccrM* family), and *RLTA1_RS09090*—were induced. Uracil-DNA glycosylase (UDG) is a critical DNA repair enzyme that is well-conserved and ubiquitous in nearly all life forms. UDG protects genomic information integrity by catalyzing the excision of uracil nucleobases from DNA, which result from misincorporation or spontaneous cytosine deamination [57]. DNA methyltransferase activity may also be vital for bacteria under acidic stress, allowing for the dynamic regulation of gene expression (e.g., stress response mechanisms, adaptation of metabolic pathways), protection, and repair of DNA, as well as the maintenance of genome integrity. These functions may collectively enhance bacterial survival and adaptation in harsh acidic environments. Like many eukaryotes, bacteria make widespread use of post-replicative DNA methylation for the epigenetic control of DNA–protein interactions [58]. The induction of gene encoding DNA ligase (*RLTA1_RS10725*) further supports the notion that DNA repair processes are crucial for bacterial survival under acid stress.

The COG K (transcription) and COG P (inorganic ion transport and metabolism) categories were both abundant in RtTA1 DEGs under acidic conditions, but gene regulation patterns varied depending on the growth medium (Figure 4). COG K contained 100 DEGs for RtTA1 grown in minimal medium and 89 DEGs in complete medium (with 36 and 32 DEGs, respectively, located in the ECR pool). These DEGs code for a large group of transcription factors (TFs) from diverse families, such as LysR [59] (the most abundant type of transcriptional regulators in prokaryotes), TetR/AcrR [60], PadR [61], GntR [62], poorly characterized RpiR/AlsR family of TFs [63] (regulating the catabolism of N-acetylmuramic acid), MerR [64] (metal-sensing regulators), MarR [65], DeoR/GlpR [66] (involved in sugar and nucleoside metabolism in diverse bacteria), global regulators of CRP/FNR [67] (e.g., regulating nitrate reductase expression) and LacI [68] families (primarily regulating carbohydrate utilization genes in response to sugar effectors), Lrp/AsnC [69] (leucine-responsive regulatory protein/asparagine synthase C products, which regulate multiple cellular processes globally (Lrp) or specifically (AsnC), including amino acid metabolism, pili synthesis, and DNA repair and recombination), and AraC/XylS [70] (a family of positive TFs with three main regulatory functions in common: carbon metabolism, stress response, and pathogenesis). In addition to classical TFs, other regulatory proteins encoded by COG K genes were differentially expressed in RtTA1 under acid stress, with the majority being upregulated. These included RNA polymerase sigma factors (e.g., sigma 70, sigma 32) and members of the GCN5-related N-acetyltransferases (GNAT) family [71]. The GNAT family is widespread in both prokaryotes and eukaryotes, and acetylation, like phosphorylation, is a major regulatory post-translational modification. It has been previously established that bacteria respond to sudden environmental changes primarily through rapid and extensive transcriptome reprogramming, while proteome changes are shifted in time [72]. Therefore, the differential expression of numerous TF-encoding genes and the upregulation of GNAT in RtTA1 under acidic stress is consistent with this adaptive strategy. Notably, a significant proportion of the differentially expressed regulators were located on ECRs, suggesting that ECR-encoded regulators contribute significantly to RtTA1’s acid stress response.

Acidic stress often leads to an influx of protons (H^+^ ions) into bacterial cells, disrupting cellular pH homeostasis. Genes in the COG P category encode proteins responsible for ion transport, including H^+^, Na^+^, and K^+^, across the cell membrane. These transport systems help expel excess H^+^ ions or bring in counter-ions to neutralize the internal pH [73]. In RtTA1, the majority of DEGs assigned to COG P were annotated as putative components of ABC transporters engaged in the transport of various ions and molecules across the membrane. The expression patterns of these genes were highly diverse, with approximately half being upregulated and the other half downregulated (Figure 4). Notably, the upregulated genes were predominantly located on ECRs. This balanced regulation of ion transporters suggests that maintaining proper transporter gene expression is critical for pH homeostasis in cells exposed to acidic stress.

The movement of ions across the membrane is closely tied to the proton motive force (PMF), which is essential for ATP synthesis. Maintaining a proper PMF is crucial for energy production, especially under stress conditions when energy demands increase [74]. In fact, we observed upregulation of the carbonic anhydrase-encoding gene (*RLTA1_RS36495*, located on pRltTA1a). Carbonic anhydrases (CAs) play a key role in regulating the levels of carbon dioxide, bicarbonate, and hydrogen ions in bacterial cells, ensuring pH homeostasis and supporting energy production [75]. Additionally, a gene coding for NAD(P)/FAD-dependent oxidoreductase (*RLTA1_RS03445*) was also upregulated. NAD(P)H-dependent oxidoreductases catalyze the oxidation or reduction of various substrates, coupled to the respective reduction or oxidation of NAD(P)H or NAD(P)^+^. These enzymes are crucial in central metabolic pathways, as NADH/NAD^+^ and NADPH/NADP^+^ serve as key redox cofactors, facilitating cellular energy balance and metabolic processes [76].

Under acidic stress, numerous downregulated genes are associated with carbohydrate transport, metabolism, and signal transduction, while some genes related to energy production pathways, such as the pentose phosphate pathway, are upregulated. This suggests that bacteria exposed to low pH prioritize energy generation and metabolic intermediates over carbohydrate transport, indicating an adaptive shift to meet higher energy demands.

### 2.10. COG and KEGG Categories for Up- and Downregulated Genes: Minimal vs. Complete Medium

Under nutrient-limited conditions, several COG categories were over-represented, with upregulated genes predominating. These categories included COG G, COG J, COG T, and COG F (nucleotide transport and metabolism). In contrast, downregulated genes dominated in COG E, COG K, COG L, COG M, and COG O (Figure 4). Additionally, COG P and COG C were strongly over-represented among the DEGs of RtTA1 grown under the tested conditions. However, the regulation pattern within these categories varied depending on the pH of the growth medium (Figure 4).

### 2.11. Limited Nutrient Availability Results in Downregulation of Genes Related to Amino Acid Transport and Metabolism, Especially Those Located on ECRs

The COG E category was strongly over-represented among the DEGs of RtTA1 during growth in minimal versus complete medium, with a predominance of downregulated genes. This was particularly evident for genes located on ECRs. For instance, at pH 7, out of the 133 DEGs in the COG E category, 53 were located on ECRs and were mostly downregulated. Regardless of pH, during RtTA1 growth in minimal medium, genes encoding putative arginase (*RLTA1_RS20750*) and ornithine aminotransferase (*RLTA1_RS20755*) were strongly downregulated. In bacteria, L-arginine serves as a precursor for various metabolites and can be a source of carbon and nitrogen. Arginase hydrolyzes arginine to L-ornithine and urea [77].

Concurrently, under acidic conditions in minimal medium, the *urtABC* gene cluster (*RLTA1_RS17340*, *RLTA1_RS17335*, and *RLTA1_RS17330*), encoding components of an ABC-type urea permease [78], was induced. Additionally, strong upregulation (log_2_FC > 5) was observed for two neighboring genes on pRleTA1d, *RLTA1_RS25275* and *RLTA1_RS25270*, encoding putative alanine racemase and D-amino acid dehydrogenase, respectively. Alanine racemase catalyzes the interconversion of L-alanine and D-alanine, while D-amino acid dehydrogenase is responsible for the oxidative deamination of D-amino acids. In *R. leguminosarum*, the primary pathway for alanine degradation involves the isomerization of L-alanine to D-alanine by alanine racemase (DadX), followed by the dehydrogenation of D-alanine to pyruvate by D-alanine dehydrogenase (DadA) [79]. Although this pathway is mainly associated with rhizobial symbiosis with plants [80], the observed upregulation suggests it may also play a role during saprophytic growth. Altogether, these findings underscore the importance of ammonia and nitrogen cycling in *R. leguminosarum*’s response to various growth conditions.

### 2.12. Numerous Genes Related to Carbohydrate Transport, Metabolism, and Energy Conversion Were Upregulated During Growth in Minimal Medium

Growth of RtTA1 in minimal medium resulted in the differential expression of many genes in the COG G category, with a total of 200 DEGs across both pH conditions. This reflects the bacteria’s need to optimize nutrient uptake, metabolic processes, and energy production in a nutrient-limited environment—a critical adaptive response for survival, growth, and competitive fitness [81]. However, several key observations regarding COG G deserve attention.

First, most DEGs in this category were upregulated during growth in minimal compared to complete medium. Second, most of these DEGs were annotated as components of various ABC transporters, including those for ribose, xylose, arabinose, galactose, maltodextrin, and other sugars. Third, ECRs significantly contributed to the pool of DEGs in COG G, with nearly half located on ECRs, particularly the pRleTA1c and pRleTA1d plasmids. These ECR-encoded genes were primarily ABC transporter-related but, interestingly, the expression pattern of these DEGs was almost evenly split between upregulated and downregulated genes.

Growing in minimal medium imposes higher energy demands, necessitating the activation of more energy-efficient pathways. The upregulation of genes related to energy production and conversion (COG C) during growth in minimal medium likely reflects the increased energy requirements for survival. For instance, the upregulation of *ccoNOG* (*RLTA1_RS23150*, *RLTA1_RS23145*, *RLTA1_RS23125*), encoding putative components of cytochrome-c oxidase as well as several ATP synthases (*RLTA1_RS20855*, *RLTA1_RS20865*, *RLTA1_RS02865*, *RLTA1_RS02850*) and ubiquinol-cytochrome C reductase (*RLTA1_RS16010*), appears to be a reasonable response to the higher energy demand.

ECR-encoded genes also contributed substantially to DEGs in COG C, especially under neutral pH conditions. For example, seven genes located on pRleTA1c (*RLTA1_RS30865*, *RLTA1_RS30780*, *RLTA1_RS30775*, *RLTA1_RS29740*, *RLTA1_RS28670*, *RLTA1_RS28665*), encoding various putative components of LLM-class flavin-dependent oxidoreductases, were strongly upregulated (log_2_FC > 4). This class of enzymes is known to participate in microbial tolerance and reduction of chromium (Cr), a form of environmental stress. In *Lysinibacillus fusiformis*, the transcriptomic response to Cr exposure resulted in a 37.4-fold (on average) induction of flavin-dependent oxidoreductase-coding genes [82].

The observed upregulation of numerous genes, including those located in the ECRs, related to carbohydrate metabolism and energy conversion aligns with the increased energy demands of cells growing in minimal medium compared to complete medium.

### 2.13. Predominant Downregulation of Transcription-Related Genes and Moderate Activation of Signaling Mechanisms as Key Elements of the Response to Nutrient Limitation

Genes in COG K represented a significant portion of RtTA1 DEGs, particularly those located on ECRs. At pH 7, 144 DEGs were identified in this category, 59 of which were ECR-encoded, while at pH 5, 65 DEGs were found, with 27 located on ECRs. While there was a clear predominance of downregulated genes in COG K, some genes were strongly upregulated. Notably, regardless of pH conditions, the growth of RtTA1 in minimal medium led to a significant upregulation (log_2_FC > 3) of a pRleTA1d-encoded putative AraC family transcriptional regulator (*RLTA1_RS25265*). The AraC family regulates various bacterial functions, including sugar catabolism, stress response, and virulence, contributing to overall metabolic versatility [70]. Additionally, a pRleTA1c-encoded putative GNAT family N-acetyltransferase (*RLTA1_RS28680*) was strongly upregulated (log_2_FC > 4.9). It is worth noticing that this gene was strongly downregulated in the response of RtTA1 to acidic pH. It may be speculated that the expression of redundant GNAT family genes may be linked to specific growth conditions, possibly to provide post-translational modifications to a defined subset of proteins (e.g., encoded by particular replicon). Indeed, several studies have highlighted that variability in acetylation may target proteins encoded by specific replicons, modulating their function or stability according to cellular needs. GNAT enzymes may play a role in modifying proteins under stress or nutrient-deprived conditions, influencing gene expression and protein activity to ensure bacterial survival and adaptation [71,83].

During growth in minimal medium, a prevalence of upregulated genes was observed among DEGs related to signal transduction (COG T). While the overall induction of these genes was moderate, there were notable exceptions. Strong upregulation (log_2_FC > 2.4) was observed for several genes encoding putative universal stress proteins (UspA), including two chromosomal genes (*RLTA1_RS23260*, *RLTA1_RS23255*) and one ECR-encoded gene (*RLTA1_RS34270*). Universal stress proteins play a critical role in bacterial adaptation to environmental stresses, including oxidative stress resistance, cell wall integrity maintenance, DNA damage repair, and regulation of cell division and growth [84].

Additionally, a pRleTA1d-encoded gene for a methyl-accepting chemotaxis protein was strongly induced (log_2_FC > 2). These proteins undergo reversible methylation during bacterial adaptation to environmental attractants and repellents, serving as a key component of bacterial chemotaxis, which is considered a protective and adaptive activity comprising a major survival strategy for bacteria [85].

Lastly, strong upregulation (log_2_FC > 3) was observed for the pRleTA1a-encoded *RLTA1_RS34280*, which codes for the putative RNA polymerase-binding transcription factor DksA. Sensing and responding to nutrient availability is a major challenge for microbial life, and DksA is a critical component of the stringent response in bacteria, also playing an essential role in the virulence of many pathogenic proteobacteria. DksA interacts with RNA polymerase (RNAP) and, together with the widely conserved master regulator guanosine-3′,5′-bisdiphosphate (ppGpp), orchestrates the global response to amino acid starvation [86]. Therefore, the upregulation of this gene in RtTA1 during growth in minimal medium seems to be a reasonable adaptive response. These results indicate that, in addition to activating the expression of genes encoding universal stress proteins, specific proteome modifications may be necessary for efficient bacterial adaptation to challenging environmental conditions.

## 3. Materials and Methods

### 3.1. Construction of the Rhizobium Pangenome

Pangenome analysis of the *Rhizobium* genus was conducted using the anvi’o software suite (version 8.1) [87]. A total of 179 complete genomes were downloaded from NCBI in June 2024 and manually curated (Supplementary Table S12). Genomic data, including both chromosomal and plasmid sequences, were processed and reformatted to create contig databases for subsequent analysis. Functional annotations were incorporated using external gene calls and functions from GenBank. Core and accessory genes were identified using Hidden Markov Models (HMMs) and classified into gene clusters based on sequence similarity. The pangenome was computed using anvi’o with stringent clustering thresholds to differentiate between core and accessory genomes. In addition to whole genomes, chromosomes and plasmids were analyzed separately to assess their respective contributions to the pangenome, and visualizations were generated to interpret the results [87].

### 3.2. Comparison of COG Category Abundance in the Accessory Pangenome

The differences in the abundance of specific COG categories in the accessory pangenome were assessed using the method outlined by diCenzo and Finan [1]. In brief, COG functional categories were assigned to genes using eggNOG-mapper [88]. For comparative analysis, the proportion of genes associated with each COG category was calculated across the accessory genomes of all replicons. These proportions were then compared to identify over- or under-represented categories in plasmids versus chromosomes, following the approach in previous studies [1,4]. Statistical significance was determined using Fisher’s exact test, and the results were visualized using custom scripts in the R statistical environment.

### 3.3. Bacterial Strain Growth Conditions

For the transcriptomic analysis, RtTA1 cells were cultivated in 79CA complex medium [89] and M1 minimal medium [90], both supplemented with 1% mannitol (Chempur, Piekary Śląskie, Poland) as the carbon source (Supplementary Table S13). The media were buffered to the desired pH using 10 mM MES (2-(N-morpholino)ethanesulfonic acid; Merck KGaA, Darmstadt, Germany) and 10 mM HEPES (4-(2-hydroxyethyl)-1-piperazineethanesulfonic acid; Merck KGaA, Darmstadt, Germany) [91]. Due to the auxotrophy of the wild-type RtTA1 strain, the minimal medium was supplemented with the following vitamins: 0.1 mg/L thiamine hydrochloride (Merck KGaA, Darmstadt, Germany), 2 mg/L calcium pantothenate (Merck KGaA, Darmstadt, Germany), and 1 μg/L biotin (Merck KGaA, Darmstadt, Germany) [90]. Growth at neutral pH (7.0) and acidic pH (5.0) was monitored by measuring the optical density at 600 nm using a Synergy H1 reader (Agilent Technologies, Inc., Santa Clara, CA, USA). For general purposes, RtTA1 were grown in 79CA medium.

### 3.4. Total RNA Isolation

High-quality DNA-free total RNA was isolated from RtTA1 cells as previously described [92,93]. Briefly, RtTA1 cells were grown in triplicate for 24 h in 79CA medium at 28 °C with shaking (200 rpm). The cultures were then diluted to an OD_600_ of 0.05 in fresh 79CA and M1 media, adjusted to pH 7.0 and 5.0, and incubated until an OD_600_ of 0.7 was reached (approximately 10^9^ CFU). Cells were harvested by centrifugation at 4 °C and immediately subjected to RNA extraction using the GeneMATRIX Universal RNA Purification Kit (EURx Sp. z o.o., Gdańsk, Poland), following the manufacturer’s protocol. Residual genomic DNA was removed using the TURBO DNA-free Kit (Thermo Fisher Scientific, Waltham, MA, USA) with rigorous DNase treatment, including minor modifications [93]. The quantity and quality of the RNA were assessed spectrophotometrically (Synergy H1 reader, Agilent Technologies, Inc., Santa Clara, CA, USA), fluorometrically (Qubit 2.0 Fluorometer with Qubit RNA High Sensitivity (HS) Assay Kit, Thermo Fisher Scientific, Waltham, MA, USA), via microcapillary electrophoresis (2100 Bioanalyzer Instrument with RNA 6000 Nano Kit, Agilent Technologies, Inc., Santa Clara, CA, USA), and through PCR analysis [92].

### 3.5. RNA Sequencing and Data Analysis

Ribosomal RNA depletion and cDNA library preparation were conducted using the Illumina Stranded Total RNA Prep with Ribo-Zero Plus kit (Illumina, Inc., San Diego, CA, USA). The samples were sequenced on the NovaSeq 6000 platform using a 2 × 150 bp paired-end sequencing mode. The raw read files for the twelve transcriptomes have been deposited in the NCBI Sequence Read Archive (SRA), and the collective data are available under BioProject accession number PRJNA1119282. Poor-quality reads and adapter sequences were removed using Trimmomatic [94]. Sequence quality was assessed with FastQC (http://www.bioinformatics.babraham.ac.uk/projects/fastqc/; accessed on 1 July 2024). Reads were mapped to the reference genome of *R. leguminosarum* bv. *trifolii* TA1 (RefSeq assembly: GCF_000430465.3) using Bowtie2 with default parameters [95], and non-uniquely mapped reads were filtered using SAMtools [96]. Normalized differential gene expression was calculated using DESeq2 [97], limma [98], NOISeq [99], and edgeR [100] software packages, all available through Bioconductor (v 3.19)—OpenSource Software for Bioinformatics (www.bioconductor.org; accessed on 1 July 2024). The following thresholds were applied to identify DEGs: an adjusted *p*-value ≤ 0.05, a log_2_FC ≤ –1 or ≥ 1 (i.e., |log_2_FC| ≥ 1.0) and Counts Per Million (CPM) ≥ 1 (to exclude genes with very low expression from the differential expression analysis). The selected DEGs were those identified consistently by all four statistical methods, based on the criteria mentioned above. Functional enrichment and annotation of DEGs were performed using reCOGnizer [101], eggNOG v5.0 [88], GSEA-pro [102], and KEGG mapper [103].

### 3.6. Exopolysaccharide Extraction and Quantification

Exopolysaccharides analyses of RtTA1 were performed as previously described [104,105]. Briefly, RtTA1 cells were grown in triplicate for 24 h in 79CA medium at 28 °C with shaking (200 rpm). The cultures were then diluted to an OD_600_ of 0.05 in fresh 79CA and M1 media, adjusted to pH 7.0 and 5.0, and cultivated with shaking for 3 days. After that, the cultures were centrifuged for 45 min at 10,000× *g*, and exopolysaccharides were precipitated from the cell-free supernatants by adding three volumes of 95% ethanol and incubating for 24 h at −20 °C. Then, the samples were centrifuged for 45 min at 10,000× *g*, and the resulting pellets were resuspended in water. The total sugar content was determined colorimetrically according to the method of DuBois et al. [106] and calculated as glucose equivalents. To estimate total protein levels, bacterial pellets were lysed by incubation in 1 M NaoH for 1 h at room temperature, followed by sonication in an ultrasonic bath. The total protein concentration was determined using the Lowry protein assay [107]. Statistical analyses were conducted on three independent biological replicates, each with three technical repeats, using R v4.2.3. Normality distribution (Shapiro–Wilk test) and homogeneity of variance (Levene’s test) were assessed to determine the appropriate statistical test. Significant differences among experimental variants were evaluated using one-way ANOVA and Tukey’s post hoc test.

## 4. Conclusions

The transcriptomic data presented in this study highlight the diverse responses of rhizobia to environmental stresses, driven by their versatile gene pool. Pangenome analysis revealed an enrichment of genes related to the transport, metabolism, and regulatory functions on ECRs, which aligns with the transcriptomic data, where numerous stress-responsive genes were found on these replicons. These results underscore that ECRs play a significant role in stress adaptation, particularly by supporting niche-specific responses and enhancing stress tolerance. Mechanisms related to central metabolism, especially those involved in ammonia production and consumption, emerged as critical strategies for coping with acidity and nutrient-limitation stress. The substantial reprogramming of gene expression, involving a considerable number of transcription factor genes also from the ECR pool as well as genes encoding enzymes potentially involved in protein modification, underscores the complexity of rhizobial adaptation mechanisms to environmental challenges. Collectively, these findings emphasize the crucial role of both transcriptomic and proteomic regulation in the survival of *Rhizobium leguminosarum* under stressful conditions.

## Figures and Tables

**Table 1:** Total number of DEGs in RtTA1 cells growing under different conditions.

Experimental Variant	Total Number of DEGs/% of Total RtTA1 CDSs	Upregulated DEGs	Downregulated DEGs
M7vC7	1700/24.2%	880	820
M5vC5	1035/14.7%	498	537
C5vC7	1129/16.0%	546	583
M5vM7	1108/15.7%	508	600

## Data Availability

The original data presented in the study are openly available in NCBI Sequence Read Archive (SRA) under BioProject accession number PRJNA1119282.

## Supplementary Materials

The following supporting information can be downloaded at https://www.mdpi.com/article/10.3390/ijms252111734/s1.
