# Supplementary material for: Transcriptomic Response of Rhizobium leguminosarum to Acidic Stress and Nutrient Limitation Is Versatile and Substantially Influenced by Extrachromosomal Gene Pool
Source: Int J Mol Sci. 2024 Oct 31;25(21):11734. doi: 10.3390/ijms252111734 (PMC11547076; doi:10.3390/ijms252111734)
Supplement: Supplementary file 1 [file ijms-25-11734-s001.zip › Supplementary Figure S1. Exopolysaccharide production by Rhizobium leguminosarum bv. trifolii TA1 under different pH conditions in complete and minimal media.pdf]

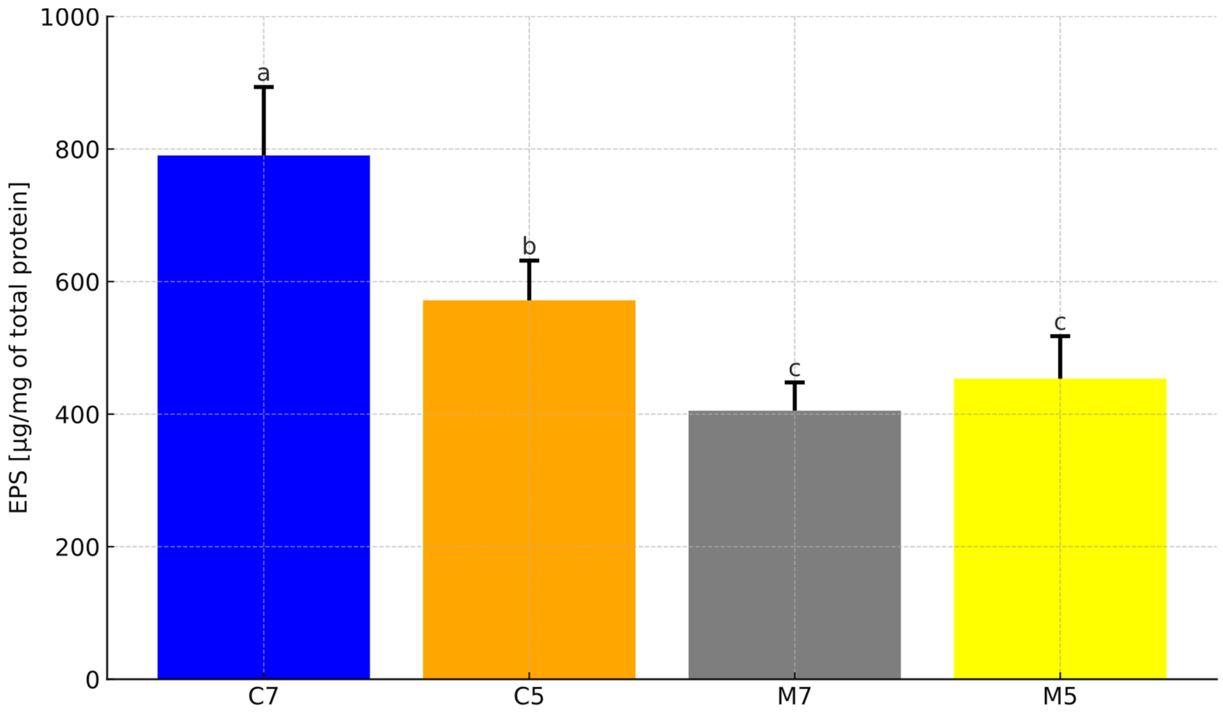

Supplementary Figure S1. Exopolysaccharide production by *Rhizobium leguminosarum* bv. *trifolii* TA1 under different pH conditions in complete and minimal media. C and M represent the 79CA complete medium and M1 minimal medium, respectively, while 7 and 5 refer to pH 7 and pH 5 of the respective medium. The EPS amount was determined by measuring the total sugar content (µg) per total protein of bacterial cells (mg) in the same culture sample. The total sugar content was quantified based on a calibration curve prepared with glucose. Bars indicate mean values from three independent experiments, each with three technical repeats. Error bars depict standard deviations. Different lower-case letters represent significant differences between groups ($F(3,22) = 52.45$, $p < 0.001$).
