# Supplementary material for: Transcriptomic Response of Rhizobium leguminosarum to Acidic Stress and Nutrient Limitation Is Versatile and Substantially Influenced by Extrachromosomal Gene Pool
Source: Int J Mol Sci. 2024 Oct 31;25(21):11734. doi: 10.3390/ijms252111734 (PMC11547076; doi:10.3390/ijms252111734)
Supplement: Supplementary file 1 [file ijms-25-11734-s001.zip › Supplementary Table S13. Composition of the bacterial media used in this study.pdf]

Table S13. Composition of the bacterial media used in this study.

Component	Concentration [g × L^{-1}]	
	79CA (complex medium)	M1 (minimal medium)
**Mannitol** [1]	10.0	10.0
**Yeast extract** [2]	1.0	–
**Acid hydrolyzed casein** [3]	1.0	–
**Calcium glycerophosphate** [4]	0.1	–
**K_2HPO_4** [5]	0.5	2.0
**NaCl** [5]	0.1	0.1
**$MgSO_4 \times 7H_2O$** [5]	0.2	0.2
**NH_4Cl** [5]	–	1.0
**KH_2PO_4** [5]	–	0.5

[1] Chempur, Piekary Śląskie, Poland

[2] A&A Biotechnology, Gdańsk, Poland

[3] Thermo Fisher Scientific, Waltham, MA, USA

[4] Merck KGaA, Darmstadt, Germany

[5] Avantor Performance Materials, Gliwice, Poland
